# Probing binding specificity of the sucrose transporter AtSUC2 with fluorescent coumarin glucosides

**DOI:** 10.1093/jxb/ery075

**Published:** 2018-03-01

**Authors:** Fabio De Moliner, Kirsten Knox, Anke Reinders, John M Ward, Paul J McLaughlin, Karl Oparka, Marc Vendrell

**Affiliations:** 1 MRC/UoE Centre for Inflammation Research, Queen’s Medical Research Institute, University of Edinburgh, UK; 2 Institute of Molecular Plant Sciences, Max Born Crescent, University of Edinburgh, UK; 3 Plant and Microbial Biology, University of Minnesota, St. Paul, MN, USA; 4 Institute of Quantitative Biology, Biochemistry and Biotechnology, Max Born Crescent, University of Edinburgh, UK

**Keywords:** Arabidopsis, coumarin, fluorescence, glucosides, imaging, phloem, sucrose, transporters

## Abstract

The phloem sucrose transporter, AtSUC2, is promiscuous with respect to substrate recognition, transporting a range of glucosides in addition to sucrose, including naturally occurring coumarin glucosides. We used the inherent fluorescence of coumarin glucosides to probe the specificity of AtSUC2 for its substrates, and determined the structure–activity relationships that confer phloem transport *in vivo* using Arabidopsis seedlings. In addition to natural coumarin glucosides, we synthesized new compounds to identify key structural features that specify recognition by AtSUC2. Our analysis of the structure–activity relationship revealed that the presence of a free hydroxyl group on the coumarin moiety is essential for binding by AtSUC2 and subsequent phloem mobility. Structural modeling of the AtSUC2 substrate-binding pocket explains some important structural requirements for the interaction of coumarin glucosides with the AtSUC2 transporter.

## Teaser

Natural and synthetic fluorescent coumarin glucosides were used as probes to analyze the key structural features that enable recognition and transport by the Arabidopsis sucrose transporter AtSUC2.

## Introduction

Phloem transport in higher plants requires the movement of sugars from mesophyll cells into the sieve element–companion cell (SE–CC) complexes in the minor veins of leaves, a process known as phloem loading (Turgeon, 2010). In those species that load sucrose from the apoplast, sucrose moves through the mesophyll in the symplasm and is subsequently transported by SWEET transporters from phloem parenchyma cells into the apoplast surrounding the SE–CC complex (Chen *et al.*, 2012; Eom *et al.*, 2015). Sucrose is then actively loaded into the SE–CC complex using one or more sucrose transporters located on the plasma membrane of the companion cell (Riesmeier *et al.*, 1994; Sauer and Stolz, 1994; Stadler and Sauer, 1996). The final entry of sucrose into the SE occurs passively through the large pore plasmodesmata that connect the CC and SE (van Bel, 1996). The vast number of molecules transported in the phloem is sufficiently large to suggest that many membrane transporters are promiscuous with regards to their substrate specificity. Recently, it was shown that glucosinolates use members of the NRT/PTR nitrate/peptide transporters to load the phloem (Nour-Eldin *et al.*, 2012), while AtSUC2, a major transporter that loads sucrose into the phloem in Arabidopsis, transports a diverse range of glucosides in addition to sucrose (Chandran *et al.*, 2003). Plasma membrane sucrose transporters in monocot species, including those responsible for phloem loading, are much more specific for sucrose and do not transport coumarin glucosides (Reinders *et al.*, 2012*a*).

Of particular utility is the fact that some of the glucosides transported by AtSUC2 are inherently fluorescent, allowing their uptake to be visualized. For example, the fluorescent coumarin glucoside, esculin, is transported into yeast cells that express AtSUC2, providing a sensitive assay for sucrose transporter function (Gora *et al.*, 2012). Recently, we showed that esculin is transported in the phloem of Arabidopsis seedlings and that it uses AtSUC2 for entry (Knoblauch *et al.*, 2015). When we replaced AtSUC2 with the barley sucrose transporter, HvSUT1, esculin was not loaded into the phloem in Arabidopsis (Knoblauch *et al.*, 2015).

Small molecule-based fluorescent probes have emerged as versatile imaging tools (Kowada *et al.*, 2015; Fernández and Vendrell, 2016; De Moliner *et al.*, 2017) and have been used to investigate the mechanism of action of drugs and xenobiotics (Er *et al.*, 2013). They represent a valuable alternative to radiotracers because of their high sensitivity, ease of utilization, and improved resolution over methods employing autoradiography (Oparka *et al.*, 1994; Knoblauch *et al.*, 2015). So far, carboxyﬂuorescein (Grignon *et al.*, 1989; Oparka *et al.*, 1994) and 8-hydroxypyrene-1,3,6-trisulfonic acid (Wright *et al.*, 1996) have been employed for this purpose, but they both emit in the green region of the visible spectrum (λ_em._ ~520 nm) where tissue autofluorescence in plants might interfere with optical imaging. Moreover, the mechanisms by which they are loaded into the phloem are only partially understood. Signal-to-noise ratios can be enhanced by using fluorophores that emit in regions of the spectra where tissue autofluorescence is minimized (Vendrell *et al.*, 2011). For instance, coumarin glucosides are useful probes as they emit in the blue region of the visible spectrum (λ_em._ ~450 nm) and enter the phloem actively using AtSUC2 (Knoblauch *et al.*, 2015). In addition, fluorescent coumarin glucosides provide a unique tool for the exploration of structure–activity relationships that underpin substrate recognition and transport by AtSUC2, potentially leading to an exploitation of AtSUC2 for the transport of xenobiotics. In the case of sucrose, the glucose moiety is the critical interacting sugar for uptake by sucrose transporters. In OsSUT1, hydroxyl positions 3 and 4 on the glucose ring have been shown to be essential for binding to arginine R188 of the carrier (Sun and Ward, 2012). An additional analysis of glucoside substrates revealed that, provided the glucose moiety is free to bind to the carrier, the non-glucose moiety (aglycon) can be relatively diverse in structure (Reinders *et al.*, 2012*a*).

Herein, we explored the effects of modifying the aglycon component of coumarin glucosides by systematically altering chemical groups in different positions of the coumarins. We examined the ability of these natural and synthetic substrates to be recognized by StSUT1 and AtSUC2 in a yeast uptake assay and by testing for phloem transport *in vivo* in Arabidopsis. In addition to known binding sites on the glucose moiety, our structure–activity relationship data show that a free hydroxyl group on the coumarin molecule is essential for substrate recognition and phloem transport.

## Materials and methods

### Chemical synthesis and characterization

Full experimental details for the synthesis of coumarin glucosides and their chemical characterization (NMR spectra) are included in Supplementary Protocol S1 at *JXB* online. Compounds with purities >95% were used for all biological studies.

### Plant material

*Arabidopsis thaliana* seeds were sterilized with 10% (v/v) bleach, rinsed once in 70% (v/v) ethanol, and then rinsed four times in dH_2_O. Seeds were plated in Petri dishes on 0.5× Murashige and Skoog medium (without sucrose), solidiﬁed with 2% (w/v) phytoagar, and stratified at 4 °C for 2 d. They were then grown for 7 d in 16 h photoperiods with 120–180 µmol m^–2^ s^–1^ at 21 °C. Seeds of *atsuc2-1* pAtSUC2:OsSUT1 were a kind gift from Jong-Seong Jeon and are described in Eom *et al.* (2016). All lines used are the ecotype Col-0.

### Loading in Arabidopsis seedlings

Seedlings were treated with a 0.3 µl droplet of 2.5% Adigor in dH_2_O (v/v) (kindly supplied by Syngenta) solution applied to each cotyledon 1 h prior to the probe application. The cotyledons were blotted gently to remove excess Adigor and then 0.3 µl of probe was applied to each cotyledon. Probes were used at 9 mg ml^–1^, apart from fraxin which was used at 5 mg ml^–1^. All were dissolved in 80% acetonitrile in dH_2_O (v/v) solution.

### Imaging

Arabidopsis roots were directly imaged on the surface of agar plates with a Leica ×2.5 HC FL Plan or ×5 HC PL Fluostar objective, using a Leica TCS SP8 confocal microscope with a HyD detector. Excitation was provided by a 405 nm solid-state laser, and emission was collected between 410 nm and 470 nm. For detailed images, a coverslip was applied directly to the root on the agar surface or the entire seedling was removed and mounted on a slide using water, with the coverslip applied to the root only to avoid contamination from the probe on the cotyledon surface. They were then imaged with either Leica ×10 HC APO w or ×20 HCX APO w objectives.

### Expression of AtSUC2 in yeast and uptake studies

The uptake of esculin and compounds **C1**–**C3** was tested in the yeast strain SEY6210 (Robinson *et al.*, 1988) transformed with either pDR196 (vector control) or sucrose transporters StSUT1 and AtSUC2 in pDR196, or OsSUT1 in pDR196/GW, as described previously for esculin (Gora *et al.*, 2012). Briefly, fluorescent compounds were added to yeast in a microtiter plate at a final concentration of 2 mM (in 25 mM NaH_2_PO_4_ buffer at pH 4), incubated at 30 ºC for 4 h, washed with buffer, and read at the appropriate wavelengths (367 nm excitation/454 nm emission for esculin and **C1**; 290 nm excitation/450 nm emission for **C2** and **C3**) using a BioTek SYNERGYMx spectrophotometer. Results are shown as relative fluorescence units. Means ±SEM (*n*=4) were calculated from four independent yeast transformants each.

### Modeling

A model for AtSUC2 was made using the Phyre2 server (Kelley *et al.*, 2015). Substrates were fitted onto the lactose analog in the structure of lactose permease (PDB Code: 1PV7) to which Phyre2 threaded the AtSUC2 protein sequence. Energy refinement was performed with Maestro using the OPLS_2005 force field (Banks *et al.*, 2005).

## Results

### Rational design of fluorescent coumarin glucosides

**Fig. 1.**
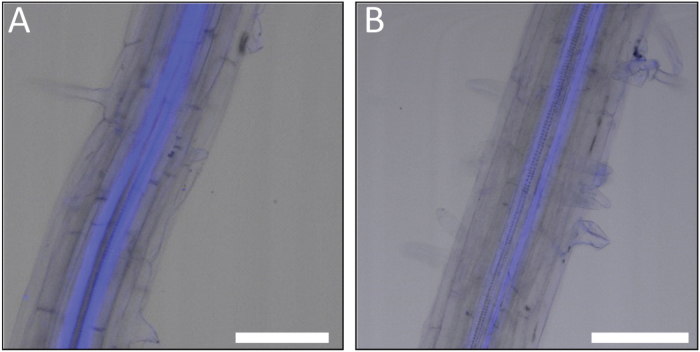
Confocal microscope images of Arabidopsis roots following application of the fluorescent coumarin glucosides esculin (A) and fraxin (B) to the cotyledons. Both coumarin glucosides are readily translocated in the phloem of the Arabidopsis root. Scale bar: 75 μm. (This figure is available in colour at *JXB* online.)

The glucosides employed in this study consist of two structural units: one conserved glucose moiety and one coumarin scaffold that is responsible for fluorescence. The two parts are connected through a β-1-linkage involving the anomeric position of the glucose and a phenolic hydroxyl group on the coumarin scaffold. While no modifications were performed on the glucose unit, coumarins with different substitution patterns on the aromatic ring were exploited. Previous work showed that coumarin aglycons were not transported into the phloem (Knoblauch *et al.*, 2015). We also demonstrated that 6,7-dihydroxycoumarin-6-β-d-glucopyranoside (esculin) and 7,8-dihydroxy-6-methoxycoumarin-8-β-d-glucopyranoside (fraxin) entered the phloem (Fig. 1; Table 1). Both compounds are phloem mobile and contain a free hydroxyl group in position 7 of the coumarin core.

**Table 1.** Coumarin glucosides evaluated as fluorescent probes to assess phloem mobility

Compound	**Chemical structure**	**Molecular weight**	**AtSUC2 activity**	**Phloem transport**
6,7-Dihydroxy-coumarin-6-β-d-glucopyranoside (esculin)	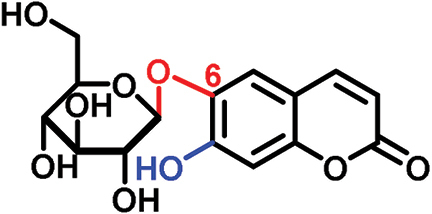	340.3	✔	✔
6-Hydroxycoumarin-6-β-d-glucopyranoside (**C2**)	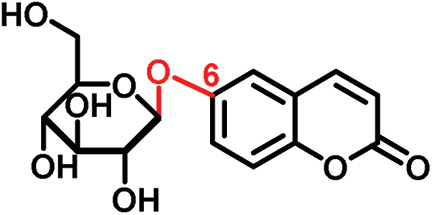	324.3	**✕**	**✕**
7,8-Dihydroxy-6-methoxycoumarin-8-β-d- glucopyranoside (fraxin)	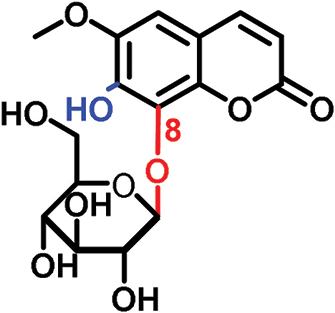	370.3	✔	✔
8-Hydroxycoumarin-8-β-d-glucopyranoside (**C3**)	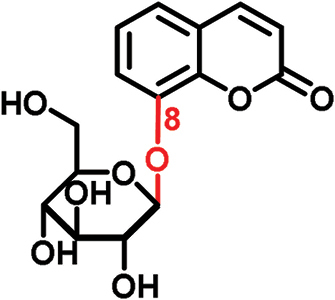	324.3	**✕**	**✕**
6,7-Dihydroxy-coumarin-β-d-glucopyranoside (cichoriin, **C1**)	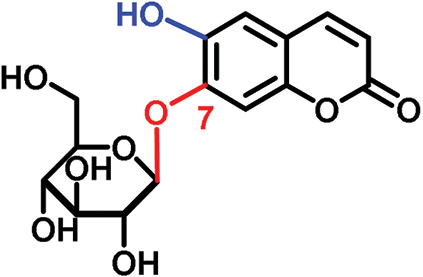	340.3	✔	✔
7-Hydroxycoumarin-7-β-d-glucopyranoside (skimmin)	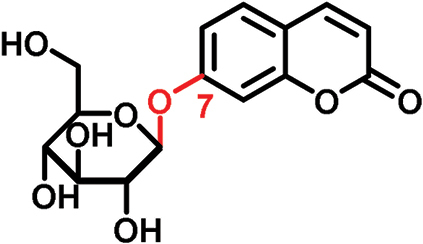	324.3	**✕**	**✕**
3-Acetyl-7-hydroxycoumarin 7-β-d-glucopyranoside	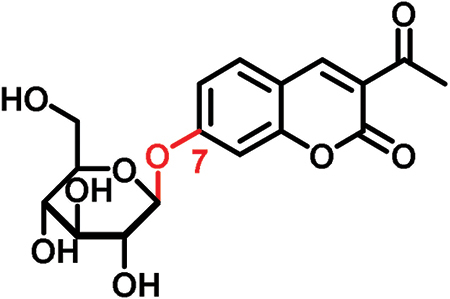	366.3	**ND**	**✕**
4-Methyl-7-hydroxycoumarin 7-β-d-glucopyranoside	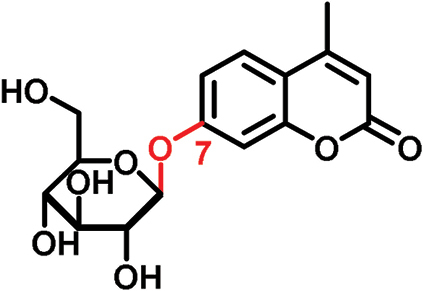	338.3	**ND**	**✕**

**Fig. 2.**
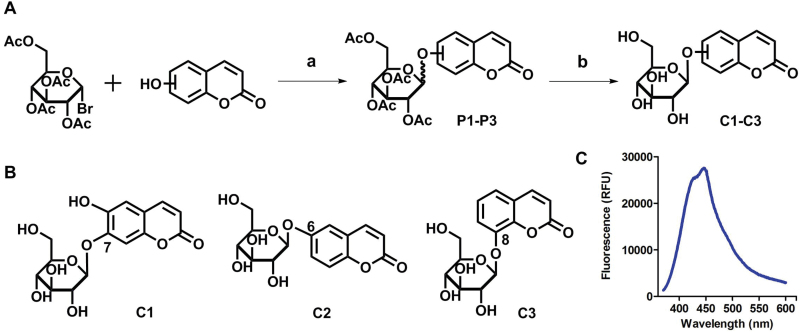
(A) General synthetic strategy for the preparation of the coumarin glucosides **C1–C3**. Reaction conditions: (a) glycosidation: KOH in acetone:H_2_O; (b) deprotection: EtONa in EtOH. (B) Structures of the fluorescent coumarin glucosides employed for biological evaluation. (C) Fluorescence spectra of cichoriin (**C1**) in aqueous media (λ_exc._: 360 nm). (This figure is available in colour at *JXB* online.)

In this study, we analyzed the role of free hydroxyl groups in coumarins as part of our structure–activity relationship evaluation. While such studies have been widely used in drug discovery and medicinal chemistry programs (Yraola *et al.*, 2004; Vendrell *et al.*, 2007; Velkov *et al.*, 2010), they have not been much exploited so far to elucidate the chemical features that enhance phloem mobility. To explore chemical diversity around this chemical group, we systematically synthesized three coumarin glucosides, namely 6,7-dihydroxy-coumarin-7-β-d-glucopyranoside (**C1**, Fig. 2), 6-hydroxycoumarin-6-β- d-glucopyranoside (**C2**, Fig. 2), and 8-hydroxycoumarin-8-β-d-glucopyranoside (**C3**, Fig. 2). While the first of these compounds retains a free hydroxyl group in position 6 and an engaged 7-position, the latter two derivatives lack the hydroxyl group and are bound to glucose in the same manner as esculin (i.e. through position 6) and fraxin (i.e. through position 8), respectively. The derivatives were assembled using a concise two-step sequence exploiting conventional synthetic processes in carbohydrate chemistry (Touisni *et al.*, 2011; Hakki *et al.*, 2015) (Fig. 2) and were fully characterized by NMR (see Supplementary Protocol S1 for full details).

### Chemical synthesis of fluorescent coumarin glucosides

α-d-Acetobromoglucose was used in all the reactions as the source of the glucose moiety. Conveniently, this monosaccharide is commercially available as a reactive bromide in the anomeric position, which enables its derivatization with nucleophiles (e.g. phenols) in basic conditions (Bjerre *et al.*, 2008; Kwan *et al.*, 2011). On the other hand, hydroxycoumarins are ideal glucoside acceptors because of their hydroxyl groups that can be deprotonated in the presence of a base to form the glycosidic bond upon displacement of the anomeric bromide (Park and Shin, 2007). Out of the three hydroxycoumarins used, 6,7-dihydroxycoumarin (esculetin, precursor of **C1**) and 6-hydroxycoumarin (precursor of **C2**) are commercially available. 8-Hydroxycoumarin (precursor of **C3**) was prepared from *trans*-2,3-dimethoxycinnamic acid by means of a Lewis acid-promoted cyclization step in the presence of boron tribromide at low temperature (Spence *et al.*, 1996).

We systematically built a series of fluorescent coumarin glucosides, using acetobromoglucose for glycosidation reactions with the above-mentioned coumarin precursors. For the synthesis, esculetin and acetobromoglucose were reacted overnight in acetone–H_2_O in the presence of potassium hydroxide (KOH). Two regioisomers were formed in a 1:1 ratio, as observed by ^1^H-NMR spectroscopy. The two fully acetylated glucosides (**P1**, Fig. 2) were separated by normal-phase column chromatography followed by deprotection in sodium ethoxide in ethanol. Compound **P1** afforded cichoriin (**C1**), the naturally occurring positional isomer of esculin, which was isolated by precipitation. Comparison with reported NMR data (Kanho *et al.*, 2004) confirmed the identity of **C1** as a single β-anomer. With this straightforward two-step protocol in place, we prepared two additional glucosides using 6-hydroxycoumarin and 8-hydroxycoumarin to obtain **C2** and **C3**, respectively. These synthetic coumarin glucosides differ in their conjugation points and the position/existence of any free hydroxyl groups. Whereas **C1** has the coumarin core connected to the glucose through position 7 of the coumarin moiety and possesses an additional free hydroxyl group in position 6 of the coumarin core, **C2** lacks any free hydroxyl group in the coumarin and forms the glycosidic bond through its position 6. **C3** is also devoid of free hydroxyl groups on the coumarin core and it is linked to the glucose through position 8 (Fig. 2; Table 1).

### Phloem mobility studies

**Fig. 3.**
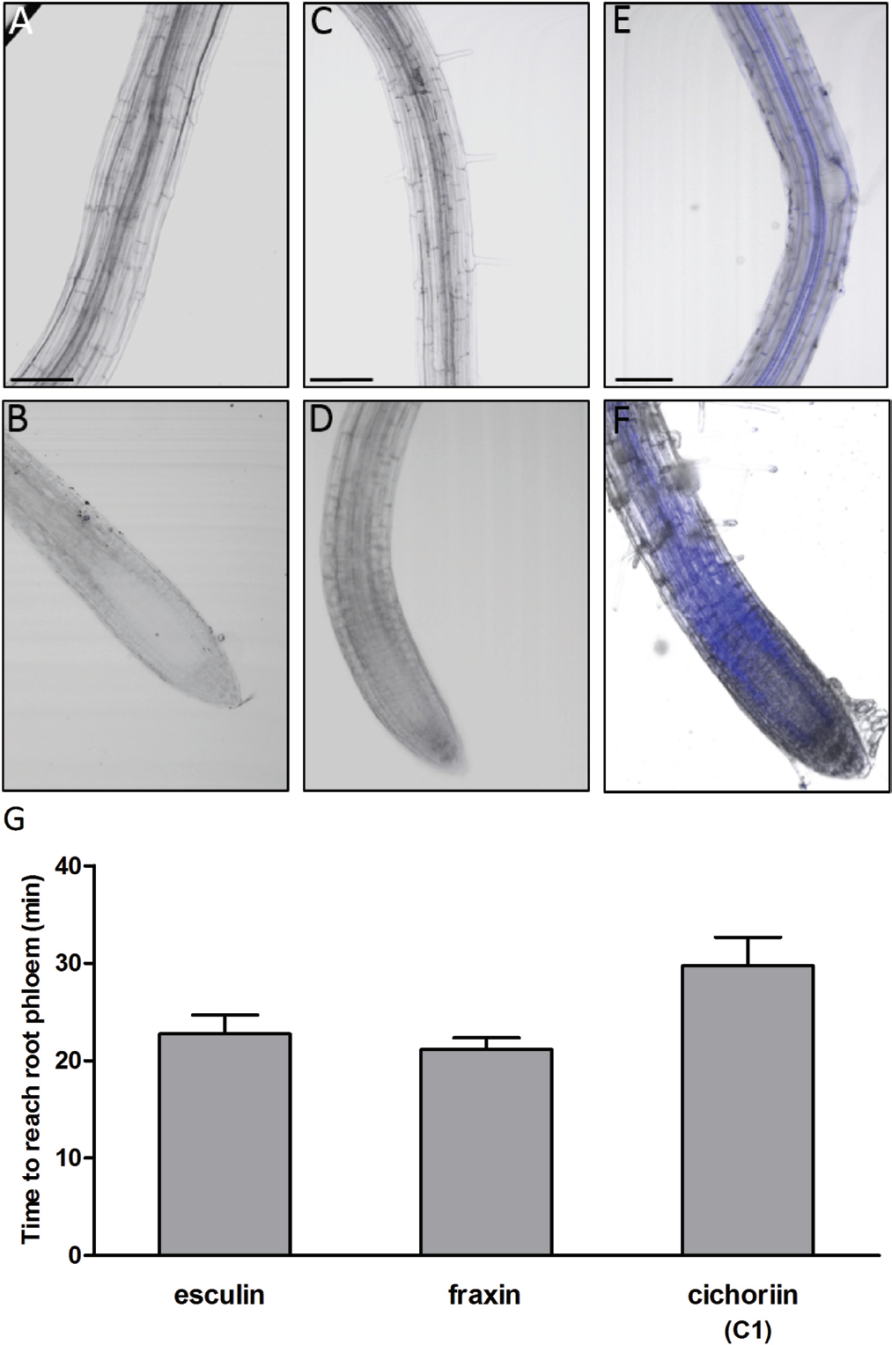
Coumarin glucosides lacking a free hydroxyl group are not translocated in the phloem of Arabidopsis. (A–F) Merged fluorescence and brightfield images of primary root (top panels) and root tip (bottom panels) after application to the cotyledons. (A and B) Compound **C2**, no translocation detected. (C and D) Compound **C3**, no translocation detected. (E and F) Compound **C1**, clear translocation in the phloem and unloading in the root tip. (G) Loading rates of esculin, fraxin, and cichoriin (**C1**) following application to the cotyledons of 7-day-old seedlings. Values are means ±SEM (*n*=26). Scale bar: 100 μm. (This figure is available in colour at *JXB* online.)

These compounds were assayed for phloem transport *in vivo* in Arabidopsis as previously described (Knoblauch *et al.*, 2015). Of all coumarin glucosides, only esculin, fraxin, and **C1** showed clear phloem mobility with similar rates (Fig. 3G), the remainder being non-mobile (Fig. 3; Table 1). Significantly, all the phloem-mobile glucosides displayed a free hydroxyl group on the coumarin moiety. Deoxy analogs of **C1** (e.g. skimmin) did not show phloem mobility, and additional modifications on the coumarin core did not rescue such a lack of mobility (Table 1). Likewise, the corresponding deoxy analogs of esculin (**C2**) and fraxin (**C3**) did not transport through AtSUC2 either, confirming the relevance of the free hydroxyl group in the coumarin core. In addition, we tested the ability of the barley sucrose transporter, HvSUT1, which does not transport esculin (Knoblauch *et al.*, 2015), to transport both natural and synthetic coumarin glucosides. We used Arabidopsis *atsuc2-5* mutants where the sucrose transporter function is rescued using HvSUT1 (Knoblauch *et al.*, 2015). In these plants, none of the coumarin glucosides was phloem mobile (Table 2). This experiment confirms that AtSUC2 and the monocot transporters HvSUT1 (Sivitz *et al.*, 2007) and OsSUT1 (Reinders *et al.*, 2012*a*) have major differences in substrate specificity, and that esculin, fraxin, and **C1** are loaded into the phloem by AtSUC2.

**Table 2.** Barley and rice sucrose transporters (HvSUT1 and OsSUT1) do not translocate coumarin glucosides when expressed in Arabidopsis lacking a functional AtSUC2 transporter, despite rescuing the mutant phenotype

	***atsuc2-5* HvSUT1** ^***a***^	***atsuc2-1* OsSUT1** ^***a***^
Esculin	<1%	0
Fraxin	<2.5%	0
Cichoriin (**C1**)	0	0

^*a*^Compounds were applied to the cotyledons of 7-day-old seedlings and the roots were monitored for 4 h to check for phloem translocation.

### Transport of coumarin glucosides by sucrose transporters in a yeast uptake assay

**Fig. 4.**
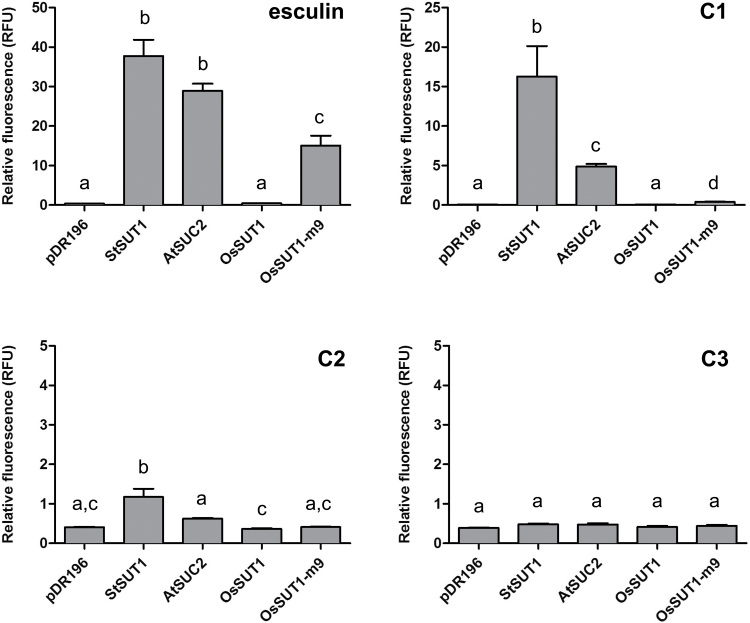
Coumarin glucoside uptake in yeast expressing AtSUC2 or homologs from potato (StSUT1), rice (OsSUT1), and the modified OsSUT1-m9. Yeast transformed with the empty vector (pDR196) was used as a negative control. The results are presented as relative fluorescence emission. Values as means ±SEM (*n*=4). Letters denote significant differences (*P*<0.05) as determined by one-way ANOVA followed by Tukey’s multiple comparison test performed on the data converted to log10.

Next, we tested the coumarin glucosides for their ability to enter yeast cells expressing different sucrose transporters, as described previously (Gora *et al.*, 2012). In these assays, the fluorescence emission was measured in yeast cells after incubation with the coumarin glucosides. Esculin uptake was observed for yeast cells expressing the potato sucrose transporter (StSUT1), AtSUC2, and OsSUT1-m9, the latter being a mutant with a nine amino acid change shown previously to transport esculin (Reinders *et al.* 2012*b*) (Fig. 4). In contrast, for esculin, the vector control (pDR196) and yeast expressing OsSUT1 only showed background fluorescence, consistent with our previous observations in Arabidopsis. Cichoriin (**C1**) uptake was observed for yeast expressing StSUT1, AtSUC2, and OsSUT1-m9, while **C2** could only be transported by StSUT1, and no significant transport was detected for AtSUC2. **C3** was not transported by any of the sucrose transporters tested (Fig. 4). The results indicate the importance of the free hydroxyl group on the coumarin moiety and the well-tolerated glycosidic bond through position 7 of the coumarin structure as two key determinants for recognition by sucrose transporters.

### Structural modeling of the AtSUC2-binding pocket

The AtSUC2 protein belongs to the major facilitator superfamily (MFS) of membrane transporters (Chang *et al.*, 2004). The most extensively studied of these is the bacterial lactose transporter, LacY, which has been crystallized and used as the basis for modeling substrate-binding sites in the transporter (Abramson *et al.*, 2003). We examined the sequences of AtSUC2 and HvSUT1, and superimposed these onto the existing LacY structural model using the Phyre 2 program (the Phyre2 web portal for protein modeling, prediction, and analysis). Out of 524 total residues, 73 residues from surface loops and distant from the substrate-binding site were not included in the model, and the final energy-refined model showed no obvious steric clashes. We validated the model by determining its Molprobity score as 1.6, which places the model’s stereochemistry in the 93rd percentile compared with other entries in the Protein Data Bank (Chen *et al.*, 2010).

**Fig. 5.**
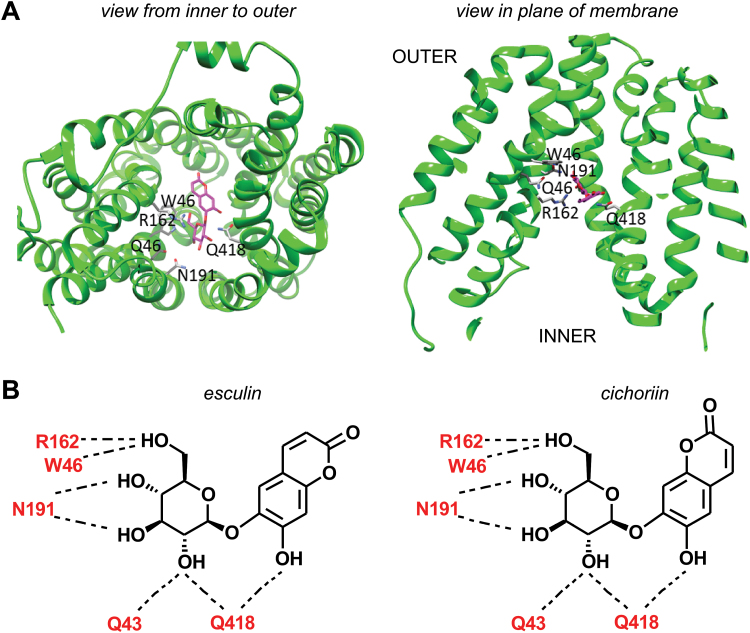
(A) Molecular model based on the LacY structure (PDB: 1PV7) showing several conserved residues (Q43, W46, R162, N191, and Q418) within potential hydrogen-bonding distance of the glucose moiety of esculin. The chemical structure of esculin is also shown. (B) Residues in AtSUC2 predicted by the model to be within potential hydrogen-bonding distance of esculin (left) and cichoriin (**C1**) (right). (This figure is available in colour at *JXB* online.)

First, we used the molecular model to analyze the interactions derived from the binding of the phloem-mobile coumarin glucoside esculin. We identified several conserved residues (e.g. Q43, W46, R162, N191, and Q418) within potential hydrogen-bonding distance of the glucose moiety of esculin (Fig. 5A). Moreover, in the model, every glucose hydroxyl group has a protein residue atom within potential hydrogen-bonding distance, which is consonant with reduced binding activities measured after substitution of each of these by a fluorine group (Hitz *et al*., 1986; Delrot *et al.*, 1991; Hecht *et al.*, 1992; Griffin *et al.*, 1993). It is also notable that the arginine residue R162 in AtSUC2 is the equivalent of arginine R188 in OsSUT1, which has been shown to be essential for sucrose transport (Sun and Ward, 2012). We also observed that the glutamine residue Q418 is within potential hydrogen-bonding distance of the glucose O2′ and also of the free hydroxyl of esculin. We employed the model to analyze the transport of our synthetic coumarin glucoside cichoriin (**C1**). The lowest energy conformer of cichoriin (**C1**) displays the coumarin moiety flipped by 180 ° when compared with esculin, leaving the free hydroxyl group in the same position relative to the protein and retaining the conformation of esculin to form potential hydrogen bonds with several residues within the transporter (Fig. 5B).

## Discussion

In plants, numerous solutes and metabolites enter the phloem for long-distance transport by carrier-mediated mechanisms. In addition, several xenobiotics enter the phloem by passive diffusion due to favorable physicochemical properties (e.g. p*K*a and lipophilicity), the latter often measured as the octanol/water partition coefficient (Hsu and Kleier, 1996). Significantly, one of the most highly phloem-mobile herbicides, glyphosate, does not have favorable physicochemical properties but enters the phloem by carrier-mediated transport (Denis and Delrot, 1993). Given the wide substrate specificity of some transporter families (Reinders *et al.*, 2012*a*; Chiba *et al.*, 2015), we envisage that substrate promiscuity could be exploited to maximize the uptake of xenobiotic compounds into the phloem. The chemical rationale of such structures requires, however, a detailed understanding of the structure–activity relationship surrounding the binding of the carrier to its substrate. In this study, we made use of the inherent fluorescence of natural and synthetic coumarin glucosides to study their interaction with the sucrose transporter AtSUC2, a protein carrier with broad substrate specificity (Chandran *et al.*, 2003). To ensure recognition by AtSUC2, glucose was always used as the sugar moiety, leaving the free hydroxyl groups at positions 3 and 4 unmodified as these are required for binding (Sun and Ward, 2012). The stereochemistry of the glycosidic bond was also maintained in its β form, as this is conserved among naturally occurring coumarin glucosides. In our previous work, we showed that some naturally occurring coumarin glucosides, such as esculin and fraxin, were phloem mobile, while others were not (Knoblauch *et al.*, 2015). Two distinct subsets of coumarin glucoside could be identified: compounds that possessed an additional free hydroxyl group on the coumarin moiety together with a glucoside bond through position 6 or 8 (i.e. esculin and fraxin, respectively), and compounds that lacked free hydroxyl groups and were connected through position 7 of different coumarin structures. Notably, only compounds belonging to the first group were found to be mobile in the phloem of Arabidopsis seedlings, making it apparent that either the free hydroxyl on the coumarin or the position involved in the glycosidic bond were key for substrate recognition by the transporter. Esculin and fraxin are transported into transgenic yeast cells and oocytes that expressed AtSUC2 (Sivitz *et al.*, 2007; Gora *et al.*, 2012). Arabidopsis *atsuc2* mutants are unable to load esculin into the phloem and, importantly, coumarins alone are not translocated unless they are conjugated to form glucoside species (Knoblauch *et al.*, 2015).

In order to determine the chemical attributes that contribute to the transport of these glucosides, cichoriin (**C1**) was synthesized and tested. **C1** displays an additional free hydroxyl group in position 6, with the coumarin being connected to glucose through position 7. Since **C1** was found to be transported into the phloem in the same manner as esculin and fraxin, we can conclude that the presence of a free hydroxyl group in the coumarin core is crucial for phloem mobility, independent of its position or the location of the glycosidic bond (i.e. glycosidic bonds at positions 6, 7, and 8 are all well tolerated). Furthermore, we extended our structure–activity relationship study to the deoxy analogs of esculin and fraxin (derivatives **C2** and **C3**, respectively) to confirm that this observation applied to coumarins with glycosidic bonds in other positions of the coumarin core. Neither **C2** nor **C3** showed phloem transport, confirming that the additional free hydroxyl group on the coumarin moiety is essential for recognition by AtSUC2. This free hydroxyl group is also present on the fructose moiety of sucrose, the natural substrate for AtSUC2, suggesting that it may also play an additional role in binding sucrose to AtSUC2.

The Phyre2 modeling program fits the AtSUC2 sequence exclusively to MFS structures in the protein data bank. A number of structures have bound glucose as well as other monosaccharides, but the model based on lactose permease (PDB code: 1PV7) is preferable for two reasons: (i) the model has a bound disaccharide (i.e. lactose analog); and (ii) the glycosidic bond lies roughly perpendicular to the channel axis. Other models that display glucose moieties in their binding sites orient the glycosidic bond roughly along the channel axis. Thus, in a disaccharide, the non-reducing end of the substrate would be more likely to make steric clashes with the transporter in one of the alternating conformations of the transport cycle. In contrast, the model based on lactose permease accommodates sucrose or other modeled substrates without any steric clashes. Moreover, as the sugar-binding site lies at the fulcrum of the ‘rocker-switch’ mechanism proposed for these transporters (Karpowich and Wang, 2008), the substrate does not change position during the power stroke of the transport cycle. Instead, the substrate experiences alternating ingress and egress routes as the protein changes around it.

The model shows potential hydrogen bonding for most of the hydroxyl groups on the glucoside binders, which is consistent with the experimental evidence that each contributes to the binding energy. The nature of the protein residues that contact the glucose moiety is characteristic of other sugar molecule-binding sites, namely that every polar glucose atom is hydrogen bonded, contains a preponderance of polar planar side chain residues [e.g. glutamine (Q), asparagine (N), and arginine (R)], and has an aromatic side chain stacked against the sugar [e.g. tryptophan (W)] (Quiocho, 1989; Taroni *et al.*, 2000). The model is also consistent with the experimental observations on the phloem mobility of different fluorescent coumarin glucosides. Notably, cichoriin (**C1**) is transported despite having a free hydroxyl on the opposite side of the coumarin core compared with esculin, since the rotation of the coumarin core places the hydroxyl groups of cichoriin in the same disposition. The free hydroxyl in both esculin and **C1** lies co-incident and is only 1.3 Å from the O6 of fructose, similarly superposed on the glucose moiety. Within the accuracy of modeling, it is possible that the free hydroxyl group forms similar hydrogen bonds to the O6 in fructose. The bond could either be with a protein residue (e.g. potentially Q418 in AtSUC2) or, in other species, with a bridging water molecule to another protein residue, which would explain why esculin and **C1** are both transported by AtSUC2. The deoxy analog of esculin (i.e. **C2**) lacks the free hydroxyl group, hence it would not bind as strongly with AtSUC2; however, it might interact more favorably with StSUT1, which has a more hydrophobic leucine (L) residue in the same position (Fig. 4). This difference could explain why **C2** transport was detected for yeast expressing StSUT1 but not AtSUC2.

**Fig. 6.**
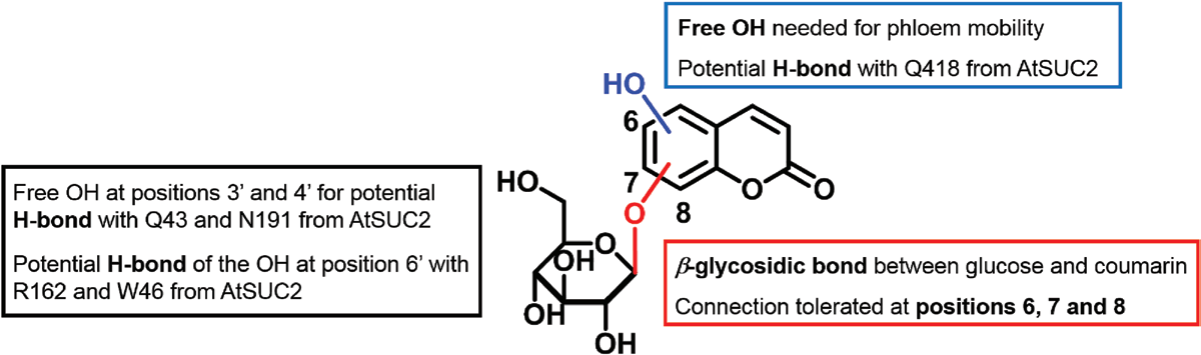
Schematic representation of the key structural modifications in coumarin glucosides that influence phloem mobility. The structure–activity relationship results are summarized to show the key points related to their recognition by AtSUC2. (This figure is available in colour at *JXB* online.)

Our model, which focuses on the binding pocket exclusively, does not fully explain the uptake specificity of other sucrose transporters or consider other factors (e.g. physicochemical properties) that might be important for phloem mobility. For instance, we measured the p*K*a of the free hydroxyl group of **C1** and observed that it is significantly more acidic than conventional phenols (i.e. compound **C1** has a p*K*a value of 8.1, whereas phenols typically show p*K*a values between 9 and 11). Therefore, the weak acidic character of **C1** might also favor its mobility (Kleier, 1988). In addition, other amino acid residues distant from the binding pocket of AtSUC2 have been shown to be required for esculin uptake (Reinders *et al*, 2012*b*). Altogether, our data demonstrate some of the requirements—including free hydroxyl groups and glycosidic bonds at different positions of the coumarin scaffold—for substrate recognition and transport. The key structure–activity relationships for binding of fluorescent coumarin glucosides to AtSUC2 are summarized in Fig. 6.

## Supplementary data

Supplementary data are available at *JXB* online.

Protocol S1. Procedures for chemical synthesis and full chemical characterization, including NMR spectra.

Supplementary Protocol
