# Supplementary material for: Probing binding specificity of the sucrose transporter AtSUC2 with fluorescent coumarin glucosides
Source: J Exp Bot. 2018 Mar 1;69(10):2473–82. doi: 10.1093/jxb/ery075 (PMC5920547; doi:10.1093/jxb/ery075)
Supplement: Supplementary Protocol [file ery075_suppl_supplementary_protocol.pdf]

SUPPLEMENTARY INFORMATION

Probing binding specificity of the sucrose transporter AtSUC2 with fluorescent coumarin glucosides

Fabio De Moliner, Kirsten Knox, Anke Reinders, John Ward, Paul McLaughlin, Karl Oparka and Marc Vendrell

Reagents

All reagents were used without further purification unless otherwise stated. HPLC-MS analysis was performed on a Waters Alliance 2695 separation module connected to a Waters PDA2996 photodiode array detector and a ZQ Micromass mass spectrometer (ESI-MS) with a Phenomenex® column (C_{18}, 5 µm, 4.6 × 150 mm). Eluents: H_2O (0.1% FA) and ACN (0.1% FA).

NMR spectra were recorded on Bruker Avance-III 500 MHz spectrometer at 308 K. Chemical shifts (δ) are reported in ppm. Multiplicities are referred by the following abbreviations: s = singlet, d = doublet, t = triplet, dd = double doublet, ddd = double double doublet, dt = double triplet, q = quartet and m = multiplet.

General procedure for the preparation of C1-C3

Hydroxycoumarin (2.5 mmol, 1.0 eq) was suspended in acetone (5 mL) and the suspension was vigorously stirred and cooled to 0°C in an ice water bath. Freshly prepared 10% w/v solution of KOH in H_2O (3 mL) was then added, followed by portion-wise addition of acetobromoglucose (2.5 mmol, 1.0 eq) over 30 min. Reaction was slowly warmed to r.t. and stirred overnight in the dark. It was then diluted with H_2O (25 mL), carefully acidified by dropwise addition of 2N HCl until pH 4-5 and extracted with CH_2Cl_2 (2 x 25 mL). Combined organics were dried over $MgSO_4$ and concentrated to dryness to give a brown oily residue that was subjected to flash column chromatography (Hexane: EtOAc 1:1) to separate unreacted starting materials and polar by-products. Protected glucosides obtained thereof were then dissolved in dry EtOH (15 mL) and a 21% w/v solution of EtONa in EtOH (0.5 mL) was added at 0°C under N_2 via a syringe. Reaction was slowly warmed to r.t. and stirred until completion (judged by HPLC-MS, 1 to 3 h). Amberlite resin was then added under vigorous stirring until pH 5-6. After filtering off the resin, the resulting solution was concentrated under reduced pressure and the final compound was obtained upon precipitation or trituration.

6,7-dihydroxy-coumarin 7-β-D-glucopyranoside (cichoriin) (C1)

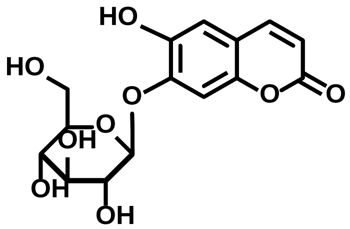

Obtained from the reaction of 6,7-dihydroxy-coumarin and acetobromoglucose upon precipitation from cold MeOH (white powder, yield over 2 steps: 27%)

^{1}H NMR (500 MHz, DMSO-d$_6$) δ 8.97 (s, 1H), 7.92 (d, *J* = 9.5 Hz, 1H), 7.14 (s, 1H), 7.10 (s, 1H), 6.30 (d, *J* = 9.5 Hz, 1H), 5.35 (s, 1H), 5.10 (d, *J* = 4.6 Hz, 1H), 5.05 (d, *J* = 5.3 Hz, 1H), 4.93 (d, *J* = 7.3 Hz, 1H), 4.63 (t, *J* = 5.5 Hz, 1H), 3.78 – 3.72 (m, 1H), 3.51 – 3.41 (m, 2H), 3.37 – 3.32 (m, 2H), 3.20 – 3.14 (m, 1H).

^{13}C NMR (120 MHz, DMSO-d$_6$) δ 161.0, 149.3, 148.3, 144.6, 144.1, 113.9, 113.5, 113.1, 103.9, 101.5, 77.8, 76.3, 73.7, 70.3, 61.2.

6-hydroxy-coumarin 6-β-D-glucopyranoside (C2)

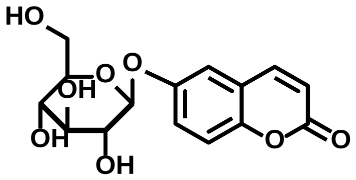

Obtained from the reaction of 6-hydroxycoumarin and acetobromoglucose upon precipitation from MeOH (white powder, yield over 2 steps: 10%)

^{1}H NMR (500 MHz, DMSO-d$_6$) δ 8.00 (d, *J* = 9.5 Hz, 1H), 7.38 (d, *J* = 2.8 Hz, 1H), 7.37 – 7.33 (m, 1H), 7.32 – 7.29 (m, 1H), 6.51(d, *J* = 9.5 Hz, 1H), 5.35 (d, *J* = 4.8 Hz, 1H), 5.09 (d, *J* = 4.6 Hz, 1H), 5.03 (d, *J* = 5.3 Hz, 1H), 4.89 (d, *J* = 7.4 Hz, 1H), 4.56 (t, *J* = 5.8 Hz, 1H), 3.74 – 3.68 (m, 1H), 3.52 – 3.46 (m, 1H), 3.38 – 3.34 (m, 1H), 3.30 – 3.22 (m, 2H), 3.21 – 3.16 (m, 1H).

^{13}C NMR (120 MHz, DMSO-d$_6$) δ 160.5, 154.1, 149.1, 144.5, 121.5, 119.6, 117.7, 117.1, 115.0, 101.5, 77.6, 77.1, 73.7, 70.1, 61.1.

8-hydroxy-coumarin 8-β-D-glucopyranoside (C3)

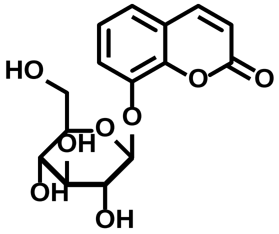

Obtained from the reaction of 8-hydroxycoumarin and acetobromoglucose upon trituration with EtOAc/MeOH (white powder, yield over 2 steps: 28%)

^{1}H NMR (500 MHz, DMSO-d_6) δ 8.07 (d, *J* = 9.6 Hz, 1H), 7.43 (dd, *J* = 8.1, 1.0 Hz, 1H), 7.35 (dd, *J* = 6.6, 1.0 Hz, 1H), 7.28 (t, *J* = 7.9 Hz, 1H), 6.52 (d, *J* = 9.5 Hz, 1H), 5.13 (d, *J* = 7.5 Hz, 1H), 4.08 (br, s, 4H), 3.68 (dd, *J* = 11.8, 1.8 Hz, 1H), 3.50 – 3.45 (m, 1H), 3.41 – 3.37 (m, 1H), 3.36 – 3.28 (m, 2H), 3.23 – 3.17 (m, 1H).

^{13}C NMR (120 MHz, DMSO-d_6) δ 160.2, 145.0, 144.6, 143.4, 124.8, 121.3, 120.1, 117.8, 116.8, 100.3, 77.6, 77.3, 73.6, 70.0, 61.1.

NMR spectra

^{1}H-NMR (C1)

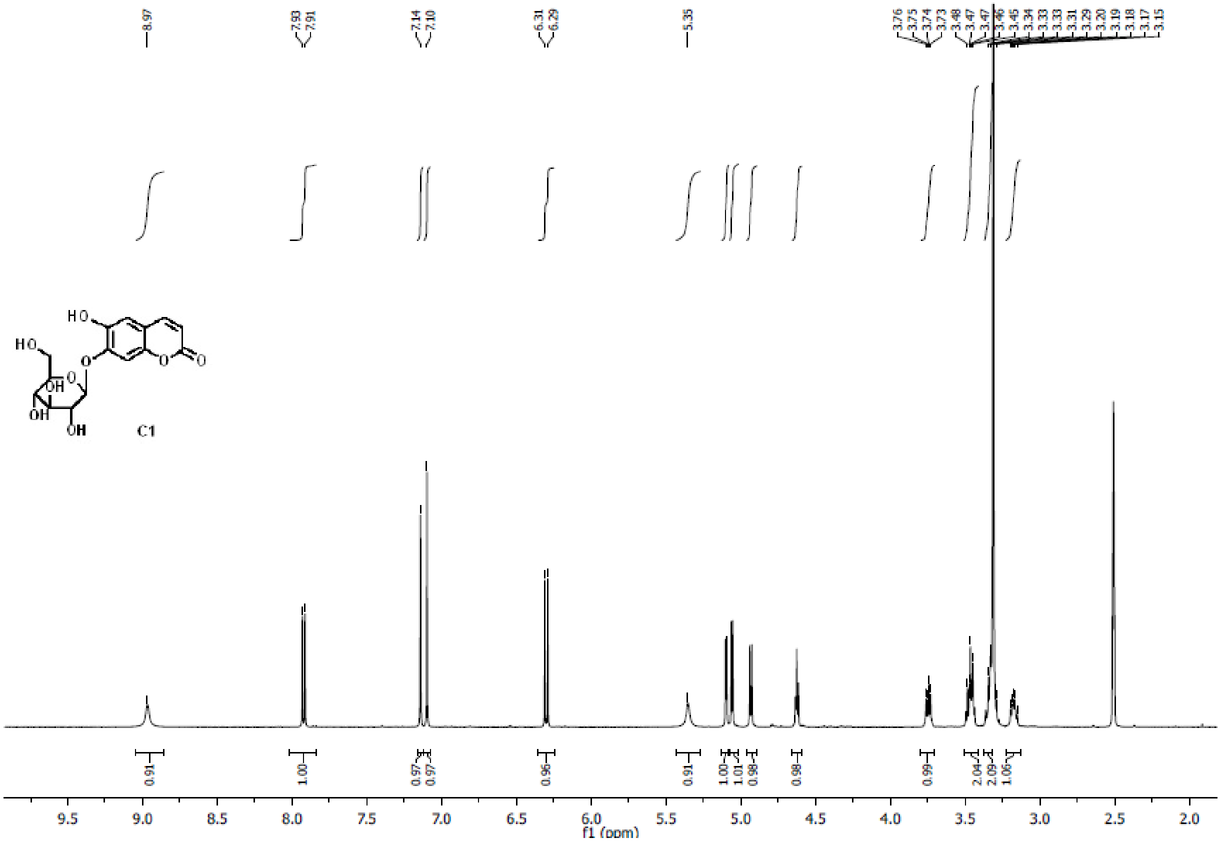

^{13}C-NMR (C1)

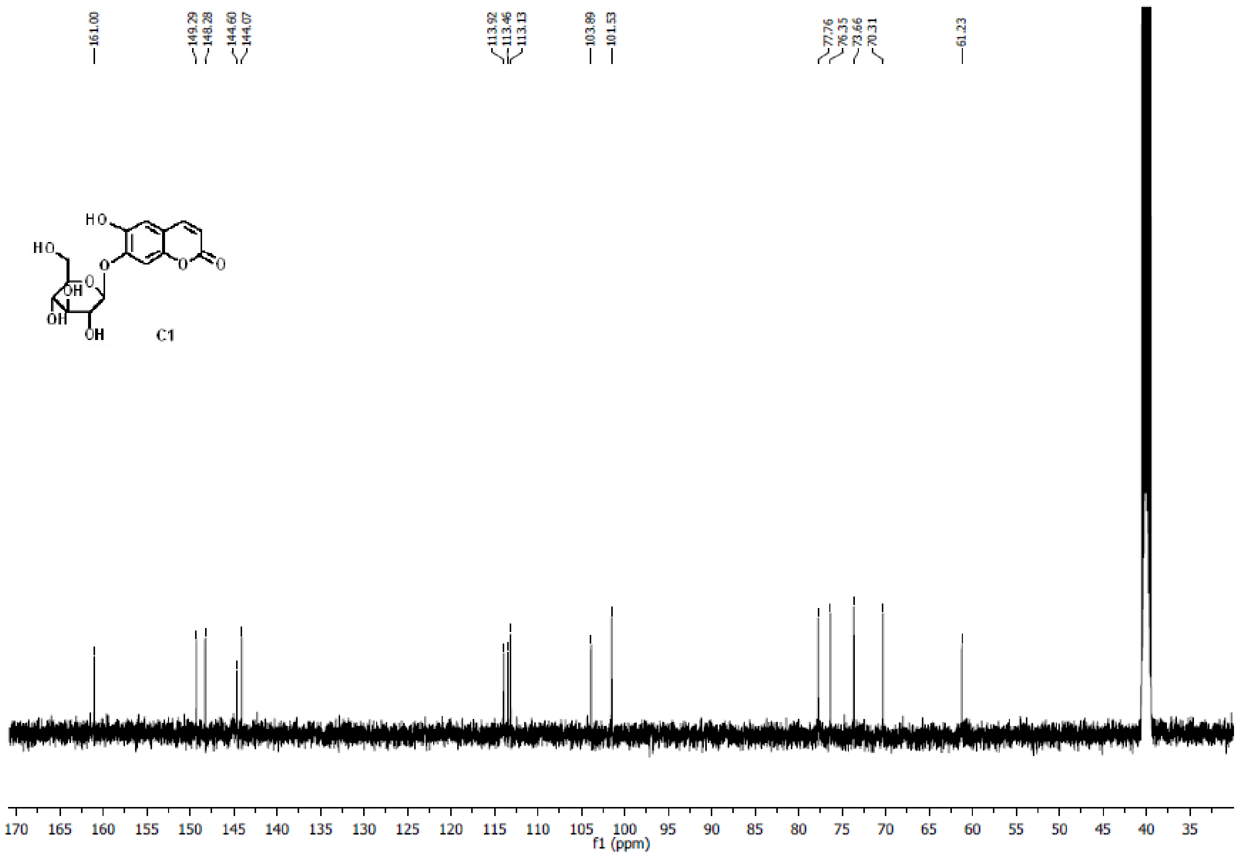
161.00
149.29
148.28
144.60
144.07
113.92
113.46
113.13
103.89
101.53
77.76
76.35
73.66
70.31
61.23
HO
HO
O
O
O
OH
OH
OH
C1
170 165 160 155 150 145 140 135 130 125 120 115 110 105 100 95 90 85 80 75 70 65 60 55 50 45 40 35
f1 (ppm)

^{1}H-NMR (C2)

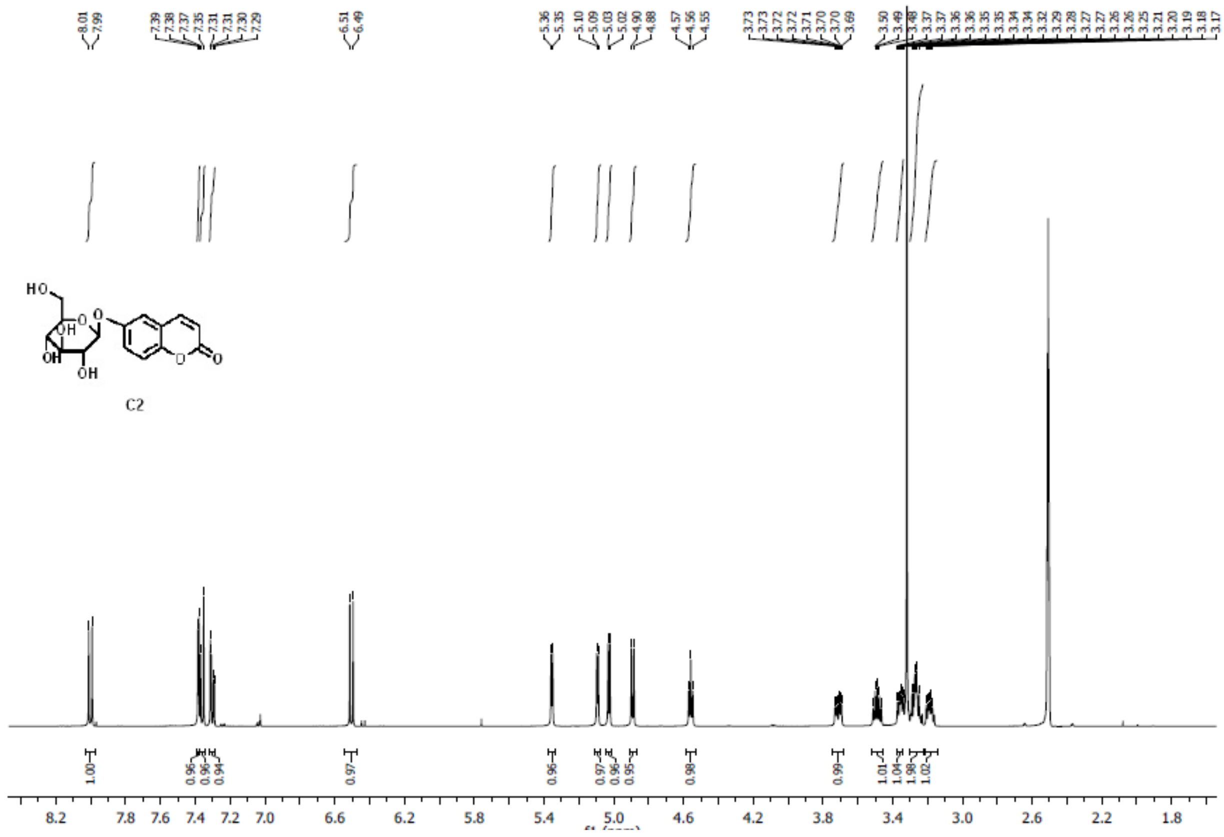

^{13}C-NMR (C2)

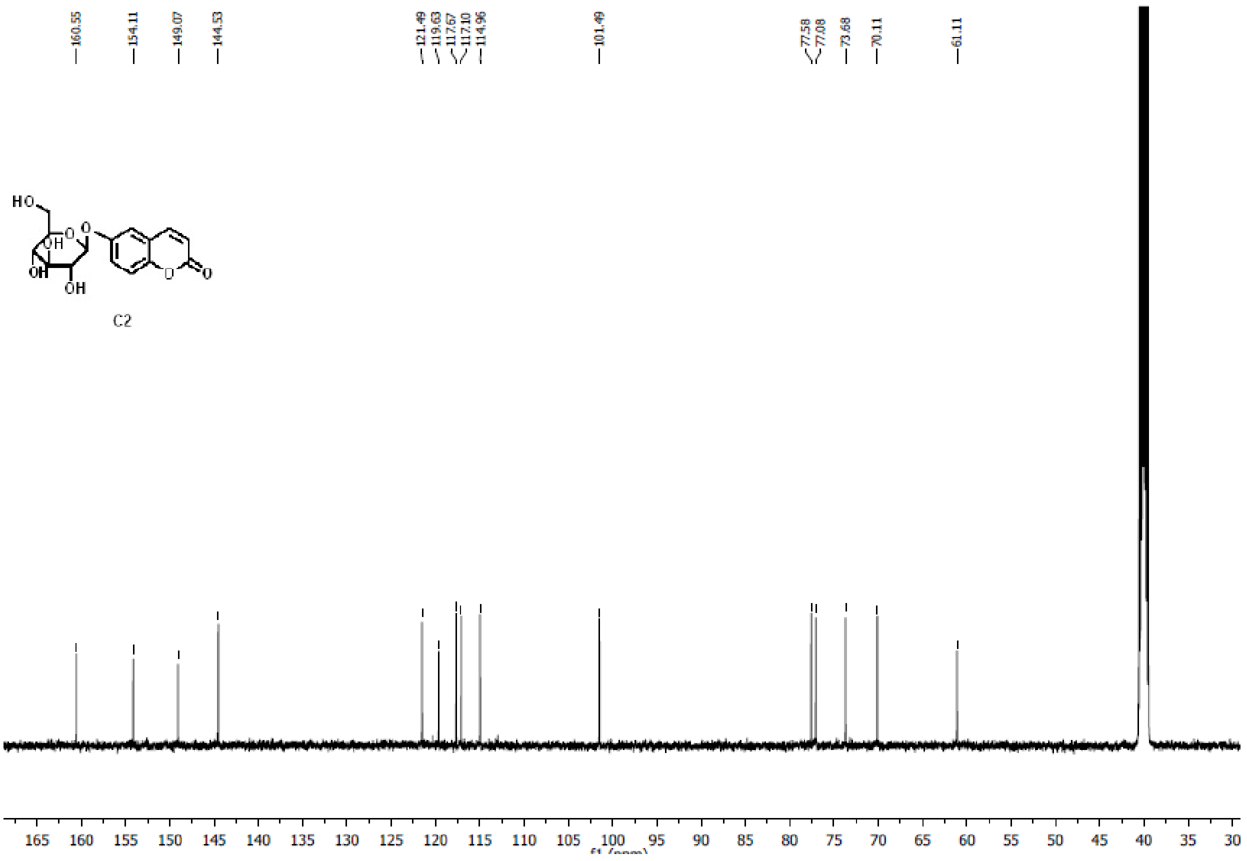

160.55
154.11
149.07
144.53
121.49
119.63
117.67
117.10
114.96
101.49
77.58
77.08
73.68
70.11
61.11
C2
165 160 155 150 145 140 135 130 125 120 115 110 105 100 95 90 85 80 75 70 65 60 55 50 45 40 35 30

^{1}H-NMR (C3)

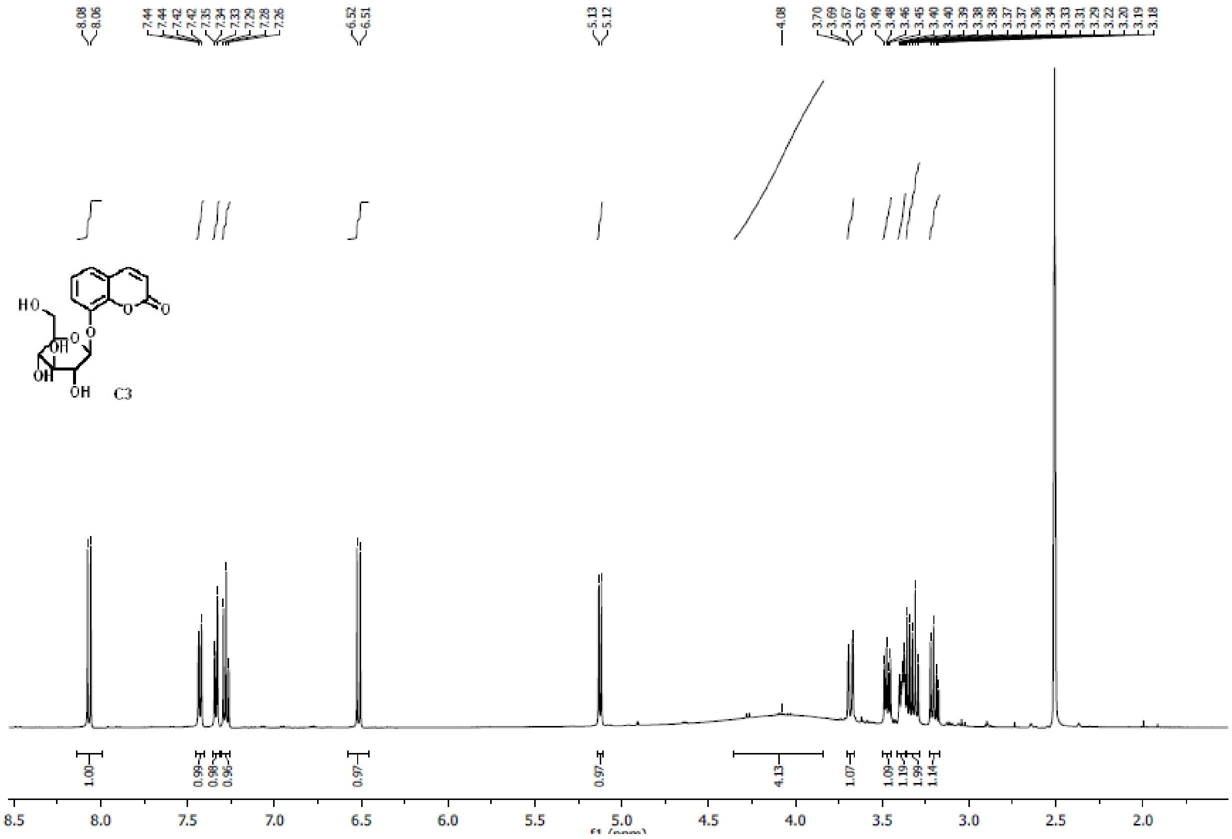

8.08
8.06
7.44
7.44
7.42
7.42
7.35
7.34
7.33
7.29
7.28
7.26
6.52
6.51
5.13
5.12
4.08
3.70
3.69
3.67
3.67
3.49
3.48
3.46
3.45
3.40
3.40
3.39
3.38
3.38
3.37
3.36
3.34
3.33
3.31
3.29
3.22
3.20
3.19
3.18
HO
O
OH
OH
OH
O
O
O
C3
1.00
0.99
0.98
0.96
0.97
0.97
4.13
1.07
1.09
1.19
1.99
1.14
8.5
8.0
7.5
7.0
6.5
6.0
5.5
5.0
4.5
4.0
3.5
3.0
2.5
2.0
f1 (ppm)

^{13}C-NMR (C3)

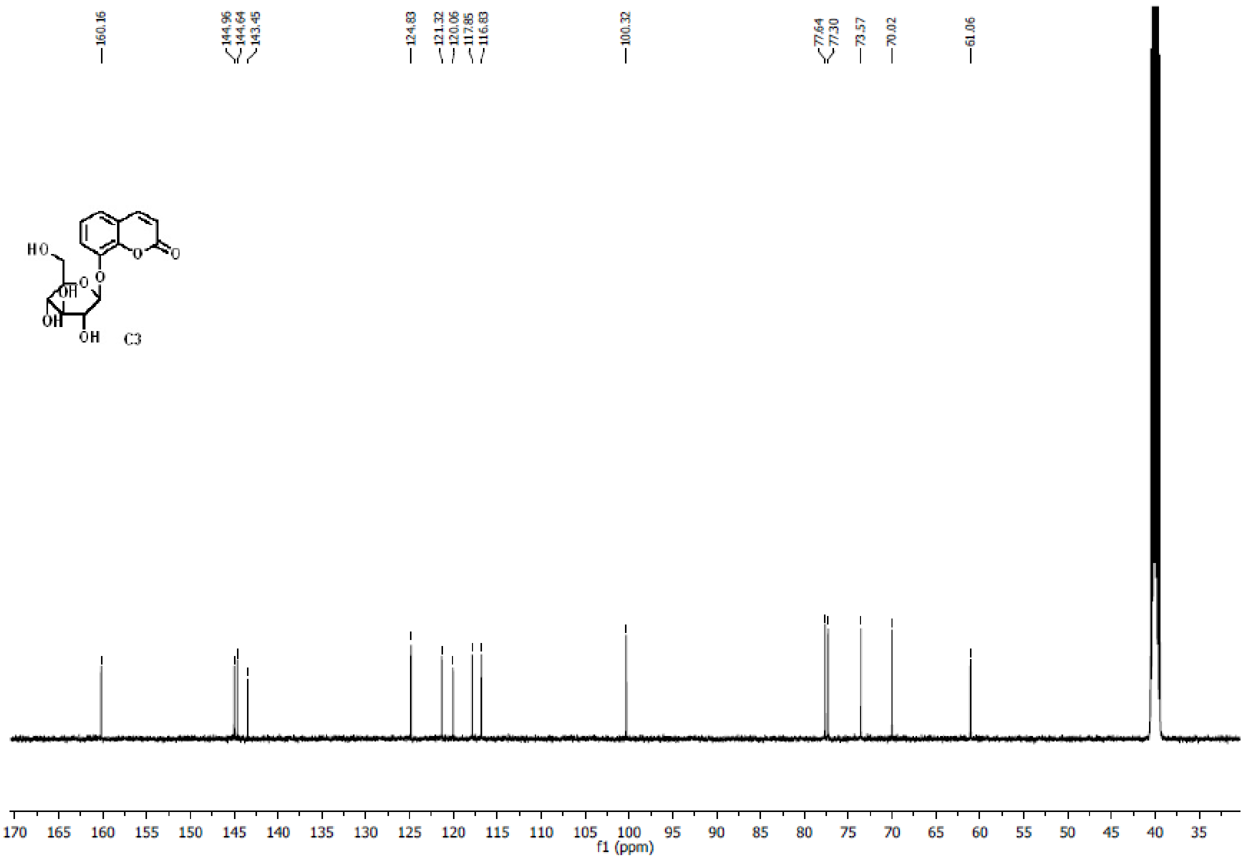

160.16
144.96
144.64
143.45
124.83
121.32
120.06
117.85
116.83
100.32
77.64
77.30
73.57
70.02
61.06
HO
O
O
OH
OH
OH
C3
170 165 160 155 150 145 140 135 130 125 120 115 110 105 100 95 90 85 80 75 70 65 60 55 50 45 40 35
f1 (ppm)
